# Multicomponent interventions for enhancing primary care: a systematic review

**DOI:** 10.3399/bjgp20X714199

**Published:** 2020-12-01

**Authors:** Geronimo Jimenez, David Matchar, Gerald Choon-Huat Koh, Josip Car

**Affiliations:** Centre for Population Health Sciences, Nanyang Technological University Singapore, Singapore.; Duke-NUS Medical School, Singapore.; Saw Swee Hock School of Public Health, National University of Singapore, Singapore.; Centre for Population Health Sciences, Nanyang Technological University Singapore, Singapore.

**Keywords:** chronic disease, health services research, healthcare reform, primary health care, systematic review

## Abstract

**Background:**

Many countries have implemented interventions to enhance primary care to strengthen their health systems. These programmes vary widely in features included and their impact on outcomes.

**Aim:**

To identify multiple-feature interventions aimed at enhancing primary care and their effects on measures of system success — that is, population health, healthcare costs and utilisation, patient satisfaction, and provider satisfaction (quadruple-aim outcomes).

**Design and setting:**

Systematic review and narrative synthesis.

**Method:**

Electronic, manual, and grey-literature searches were performed for articles describing multicomponent primary care interventions, providing details of their innovation features, relationship to the ‘4Cs’ (first contact, comprehensiveness, coordination, and continuity), and impact on quadruple-aim outcomes. After abstract and full-text screening, articles were selected and their quality appraised. Results were synthesised in a narrative form.

**Results:**

From 37 included articles, most interventions aimed to improve access, enhance incentives for providers, provide team-based care, and introduce technologies. The most consistent improvements related to increased primary care visits and screening/preventive services, and improved patient and provider satisfaction; mixed results were found for hospital admissions, emergency department visits, and expenditures. The available data were not sufficient to link interventions, achievement of the 4Cs, and outcomes.

**Conclusion:**

Most analysed interventions improved some aspects of primary care while, simultaneously, producing non-statistically significant impacts, depending on the features of the interventions, the measured outcome(s), and the populations being studied. A critical research gap was revealed, namely, in terms of which intervention features to enhance primary care (alone or in combination) produce the most consistent benefits.

## INTRODUCTION

Primary care has been identified as a possible solution to address the challenges imposed on health systems by changing demographics, an increasingly ageing population, and the rise of the global burden of non-communicable chronic diseases.^1^^–^^6^ International evidence suggests that health systems with strong primary health care produce better and more equitable health outcomes, are more efficient, and can achieve higher user satisfaction when compared with health systems that have only a weak primary care orientation.^7^

Primary care has been defined as *‘the provision of integrated, accessible healthcare services by clinicians who are accountable for addressing a large majority of personal healthcare needs, developing a sustained partnership with patients, and practicing* [sic] *in the context of family and community’*.^2^ From this, the four main functions of primary care services were established; referred to as the ‘4Cs’ of primary care, these are:

- first-contact access for each need;
- longitudinal person-centred care (continuity);
- comprehensive care for most health needs; and
- coordinated care when it must be sought elsewhere.

As a result, primary care is assessed according to how well these four functions are fulfilled.^2^^,^^6^

Primary care systems have had to evolve in order to address new challenges. Efforts have been deployed at policy level in many countries including the US,^8^ Spain,^9^ the Netherlands,^10^ Canada,^11^ and the UK,^12^ which, in turn, has led to the implementation of a variety of programmes or innovations on the ground, with the objective of enhancing primary care. This study aimed to identify:

- multicomponent interventions or ‘innovation environments’ — locations in which a comprehensive effort was initiated to enhance primary care; and
- their effects on the ‘quadruple-aim’ outcomes (population health, healthcare costs and utilisation, patient satisfaction, and provider satisfaction^13^).

**Figure 1.**
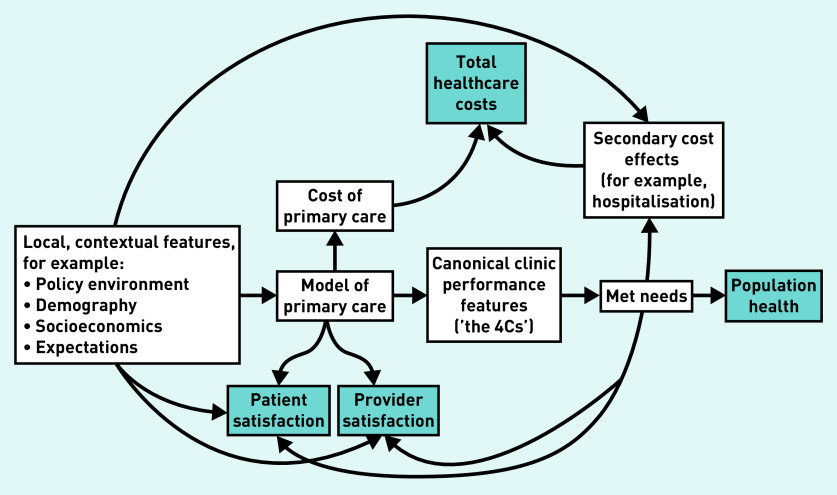
**Conceptual framework linking contextual features, primary care characteristics in terms of the ‘4Cs’, and system performance (the ‘quadruple-aim’ outcomes).**

To guide this review, the authors developed a conceptual framework, built around Starfield *et al*’s 4Cs,^2^ which are central to a clinic’s ability to meet patient needs and, thus, improve population health; the framework is shown in Figure 1. For each identified effort to enhance primary care, the key questions were:

- which of the 4Cs were targeted;
- how did it impact any of the quadruple-aim outcomes; and
- how was the success of the effort related to the specific features of the model of care (that is, details of how change was implemented) and contextual factors (that is, policy, demographics, socioeconomics, and so on).

**Table:** How this fits in

Many countries have implemented multicomponent interventions — that is, strategies composed of several innovation features — to enhance primary care as a way of strengthening their health systems to cope with an ageing population, the rise of chronic conditions, and increased healthcare costs. The number and types of features these strategies include, their impact on the primary care core functions (the 4Cs — first contact, comprehensiveness, coordination, and continuity), and their effect on population health, healthcare costs and utilisation, and patient and provider satisfaction, have not been explored. This study identified the most common features included in these interventions, while connecting them to the 4Cs and their impact on outcomes. Most interventions improved some outcomes more consistently than others, such as increasing primary care visits in relation to specialist visits, increasing preventive and screening services, and improving provider satisfaction. However, at the same time, they produced mixed results for most of the remaining outcomes — most notably for hospital admissions, emergency department visits, and expenditures. This signals a need to identify the best combination of features that would produce the most consistent benefits for various populations, policy environments, and health system structures. The results identified here can provide valuable insights to clinicians and primary healthcare system administrators designing multicomponent interventions to enhance primary care.

## METHOD

The method for this review was developed based on the criteria for conducting systematic reviews in the *Cochrane Handbook of Systematic Reviews of Interventions*^14^ to ensure methodological rigour and minimise the risk of bias. The main research question and a description of the elements are given in Box 1.

**Box 1.** Main research question (PICO format) and description of elements

Research question: In primary care settings (P), how do multicomponent enhanced primary care interventions (I), compared to usual care (C), affect the quadruple-aim outcomes (O)?

Population (P): adult patients requiring primary care, and/or adults with chronic conditions in primary care settings, general practice, and family medicine (including community settings) Interventions (I): multicomponent primary care interventions as a whole ‘package’ that may include policy and/or financing changes, organisational restructuring, manpower changes, service delivery interventions, technology interventions, and so on, and were implemented within a particular jurisdiction Comparator (C): usual care (that is, comparison with the situation before the implementation or intervention took place in the same jurisdiction pre-post-evaluation), or comparison with a similar jurisdiction that has not gone through a change and so on) Outcomes (O): any or all of the four outcomes in the quadruple aim, where studies must have reported numerical values/magnitudes of changes in outcomes

### Search strategy and inclusion/exclusion criteria

A search strategy was developed (Supplementary Box S1), which included three main-term packages:

- primary health care-related terms;
- innovation/enhancement/reform-related terms; and
- types of studies to be included.

The search was performed on 30 May 2019, by one author, in Ovid MEDLINE. There were no year or language limits. In addition to the electronic database search, references of included studies were manually searched for relevant articles, and a grey-literature search (using http://www.opengrey.org) was performed on 11 December 2019, using the terms ‘primary care’ and ‘intervention’.

Studies were included if they:

- reported an effort to improve primary care;
- were clinical trial/randomised controlled trials, evaluations, comparative studies, intervention studies, effectiveness studies, observational studies, or case–control studies;
- explicitly mentioned that the effort aimed to improve any of the 4Cs, and/or provided sufficient description to derive a ‘C’ of primary care being influenced; and
- reported on any of the quadruple-aim outcomes (population health, healthcare cost/utilisation, patient satisfaction, provider satisfaction), and provided a magnitude/numerical value.

Studies were excluded if:

- the study topic was not about primary care, or was about primary care but focused on a sub-area not relevant for this study (for example, maternal health, paediatric populations, dental health, hospital-based studies, alcohol/substance misuse, smoking, screening/risk assessment, Aboriginal/indigenous populations, US armed forces, immigrants);
- they were reviews, qualitative, protocols, validations, guidelines, or perspectives/editorials. (Some studies that were not interventions could still provide valuable information that was applicable to what was needed and, as such, relevant reviews were selected so that their references could be checked.);
- a primary care ‘C’ could not be identified as having influenced the effort/intervention of the study;
- they did not report a numerical magnitude of a change for any of the quadruple-aim outcomes, or they measured non-relevant outcomes (for example, inappropriate prescribing/amount of prescribing, referral rates, weight loss, alcohol use/number of drinks per week).

### Hierarchy classification and study selection

Given the search strategy and specific needs for the articles to be included, it was expected that a large number of partially suitable studies would be retrieved. Accordingly, the authors performed a hierarchy classification, similar to that performed by McCrory *et al*.^15^ Studies were classified into three main categories:

- Tier 1 (most suitable and useful): studies clearly describing the implementation of multicomponent interventions and their elements (with at least three innovation elements); reporting clearly on relevant quadruple-aim outcomes and providing magnitudes (for at least five outcome measures); influencing at least one of the 4Cs;
- Tier 3 (least suitable): very specific efforts to enhance a minor, isolated aspect of primary care; non-multicomponent; reporting on a specific outcome; and
- Tier 2: less specific than Tier 3 articles, but not as comprehensive as Tier 1 articles (that is, not having at least three innovation elements and not reporting on at least five relevant outcome measures).

A priori studies in Tier 2 were to be evaluated and included in the analysis on a needs basis, in case few Tier 1 articles were available. (A full list of Tier 2 and Tier 3 articles is given in Supplementary Box S2).

The screening, study selection, and hierarchy classification were performed by one author. Study selection and hierarchy classification were then iteratively spot-checked by the other three authors in several rounds. Ten of the 37 selected studies were read in full and analysed in depth by all the authors at convened consensus meetings to ensure methodological quality and relevance for the project’s needs.

### Quality evaluation

Study quality was evaluated using the National Institutes of Health — National Health, Lung, and Blood Institute’s study quality assessment tools (https://www.nhlbi.nih.gov/health-topics/study-quality-assessment-tools); this set of tools is able to cover a wide range of study designs and was suitable given the heterogeneity of the analysed studies. The quality evaluation process, rather than being used to exclude articles of lower quality, was used to guide on the reporting of outcomes and to identify the evidence that derived from studies with a stronger design that were, therefore, of better quality. Quality ratings used were ‘good’, ‘fair’, and ‘poor’. The quality evaluation tools and results are detailed in Supplementary Box S3.

### Data extraction, reporting of results, and analysis

Data extraction was performed using a pre-established form that included articles’ general information, study characteristics, the 4Cs being influenced, and the impact on quadruple-aim outcomes (Supplementary Table S1).

The data are presented using a narrative, descriptive approach, which is typically used when the research question dictates the inclusion of a wide range of research designs, including qualitative and/or quantitative findings.^16^ From the data, the authors derived the most recurrent outcomes that had statistically significant benefits and disadvantages for primary care, as well as those that did not show statistical significance or involved mixed results; this was done to present the innovation environments that were associated with the corresponding outcomes. These are summarised in the text, Supplementary Tables S2a–d, and Supplementary Table S3, and are presented in detail in Supplementary Table S4. Given the heterogeneity of the included studies and their interventions, it was not possible to perform a meta-analysis.

## RESULTS

### Search results

The electronic database search resulted in 2018 individual articles. After title and abstract screening, 1770 articles were excluded and 248 were read in full. Subjecting the full-text articles to the inclusion and exclusion criteria resulted in an additional 36 studies being excluded. The hierarchy classification was conducted on the remaining 212 studies; of these, 76 were too specific and classified as Tier 3 articles; 101 were, arguably, more relevant and grouped into Tier 2 articles; and 35 were highly relevant and classified as Tier 1. Given that this number was sufficient for acquiring numerical parameters of the effects of the multicomponent interventions, Tier 2 articles were neither assessed nor included for subsequent analysis.

**Figure 2.**
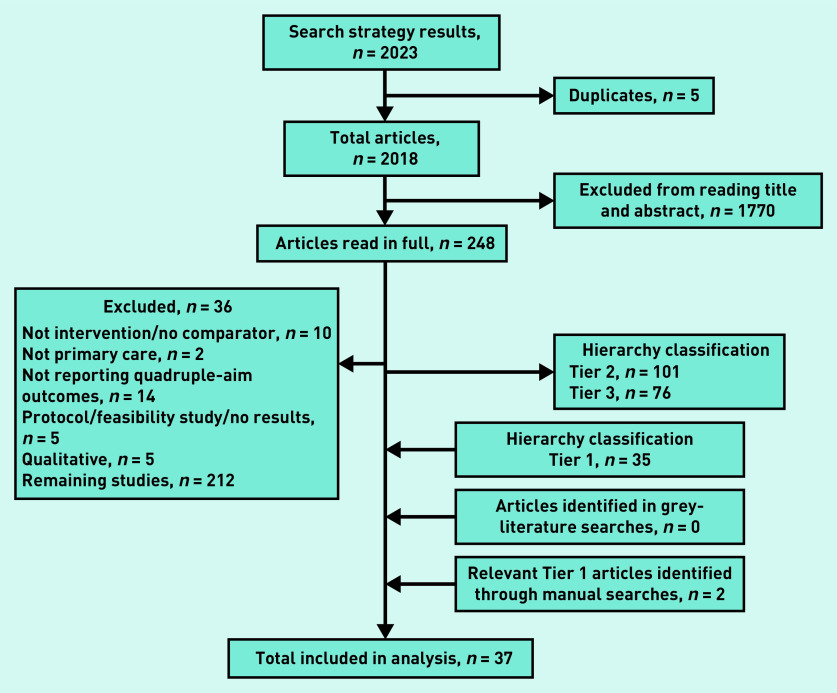
**PRISMA diagram of the study selection process.**

A manual search through the references of these Tier 1 studies resulted in the addition of two more articles. The grey-literature search resulted in no additional relevant articles, bringing the total number of studies included in the analysis to 37.^17^^–^^53^ The selection process is detailed in Figure 2.

### Study characteristics

A summary of the characteristics of the included studies is outlined in Table 1. (For full details on types of interventions found in each study see Supplementary Table S5.) Publication years ranged from 1999 to 2018; 67% of the articles had been published since 2013 and most derived from developed, high-income countries.

**Table 1.** Summary of studies’ (*n* = 37) characteristics

**Characteristic**	***n* (%)**
Publication years	
1999–2009	5 (14)
2010–2012	7 (19)
2013–2015	12 (32)
2016–2018	13 (35)

Countries	
US	23 (62)
Canada	6 (16)
Germany	2 (5)
Spain	2 (5)
France	1 (3)
The Netherlands	1 (3)
Argentina	1 (3)
Mexico	1 (3)

Policies/programmes influencing interventions	
PCMH/ACA	9 (24)
Medicare/Medicaid	3 (8)
Family Medicine Group/Network	4 (11)
National/regional policies	3 (8)
Others	2 (5)
No policies mentioned	16 (43)

Study designs	
Controlled interventions	11 (30)
Controlled observational cohorts/cross-sectional	16 (43)
Case–control	1 (3)
Uncontrolled pre-post	9 (24)

Study quality	
Good	13 (35)
Fair	19 (51)
Poor	5 (14)

Patient population	
General patients/enrolled in programme	15 (41)
Chronic condition patients	10 (27)
Special populations	10 (27)
No patients	2 (5)

Types of outcomes studied^a^	
Population health	15 (41)
Healthcare costs/resource utilisation	28 (76)
Patient satisfaction	6 (16)
Provider satisfaction	3 (8)

a Non-exclusive category. ACA = Affordable Care Act. PCMH = patient-centred medical home.

In 21 of the 37 included studies, policies or government programmes influencing the implementation of primary care interventions were mentioned. Those articles that did not explicitly mention a policy cited specific health problems in their jurisdictions, special populations that needed health-related support, and/or the need to curb health-related costs and improve outcomes as the reasons for implementing interventions. Populations being studied could be divided into three main groups: general patients enrolled in the practice or programme being studied; chronically ill patients; and special populations (such as older people, those with frailty, or ‘superutilisers’).

The quality assessment exercise by study design resulted in:

- five ‘good’ and six ‘fair’ controlled interventions;
- five ‘good’, nine ‘fair’, and two ‘poor’ observational cohort and cross-sectional studies with controls;
- one ‘fair’ case study; and
- three ‘good’, three ‘fair’, and three ‘poor’ pre-post-studies with no control (Supplementary Box 3).

The most commonly reported type of outcome was healthcare costs and resource utilisation (in 28 studies), followed by population health outcomes (in 15 studies), patient satisfaction (in six studies), and provider satisfaction (in three studies) (Table 1 and Supplementary Table S5). However, these are not mutually exclusive, as 25 studies reported on one type of outcome only, but nine reported on two, and three reported on three types of outcomes.

### Descriptions of interventions, impact on the 4Cs, and their effects on outcomes

The characteristics of the intervention programmes or sets of innovation features described in the articles varied widely, but it was possible for the authors to group the vast majority of innovations into 18 distinct (and one non-distinct) categories (Table 2).

**Table 2.** Primary care innovation categories and definitions

**Category**	**Definition**
Accountability mechanisms	Programmes/systems to identify a population for which a primary care provider/practice was responsible for (for example, empanelment, registries, incentives to enrol patients)
Care plan development	Creation of plans for patient care
Case/care management	Innovations that include case-management fees or include the addition of a case manager (for example, risk-stratified case management)
Efforts to improve performance monitoring/appraisal	Programmes/systems that added or changed quality measures, or the way these were measured and identified
Enhanced continuity/transition-based efforts	Programmes/systems designed to follow up with patients or support in transitioning through different care levels (for example, routine monitoring to identify changes in patients’ conditions, transition coaches)
Enhanced coordination/information exchange efforts	Systems designed to improve the coordination and information exchange between different levels of care (for example, care coordination fees, enhanced referral systems)
Enhanced service capacity	Innovations aimed at expanding the services provided at a primary care site (for example, equipping a primary care clinic to handle emergencies, adding geriatric services, adding preventive care services)
Improved access	Systems facilitating access to primary care services (that is, expansion of service hours, telephone/internet access, home visits, and so on)
Improved patient self-management/engagement	Programmes/innovations aimed at engaging patients/caregivers in their own care (for example, education or coaching, shared decision making)
Improved specialty care access/support	Innovation aimed at facilitating access to specialists (for example, removal of primary care gatekeeping, adding specialists to primary care clinic)
Inclusion of new/enhanced roles	Adding new roles to the primary care practices (for example, healthcare assistants, practice facilitators) or enhancing existing roles (for example, nurse acting as care manager)
Increased control of workload	Enhancements aimed at alleviating physicians’ caseloads by shifting activities to other team members
Payment-based enhancements	Innovations related to changing the way providers get paid, including monetary incentives and compensation formulas (for example, fee-for-service versus capitation versus pay-for-performance)
Pharmacy/medication-related efforts	Programmes related to improving pharmacy or medication prescription, use of IT pharmacotherapy tools, efforts to avoid duplicate medications, and so on
Provider education or training	Programmes aimed to improve primary care services by educating or training primary care health professionals
Social or community services engagement	Systems aimed at engaging community-based or social services
Team-based care	Systems in which care is provided by a team of providers
Technology enhancements	Innovations in which a technology was introduced to improve services (for example, shared electronic medical records across different providers, IT system for data-driven improvements, online tools for a variety of enhanced capabilities)
Others	Innovations not classified in other categories that include: alternative medicine initiatives, enhanced screenings, redesign of service/organisational interventions to reduce variation in physician productivity

**Figure 3.**
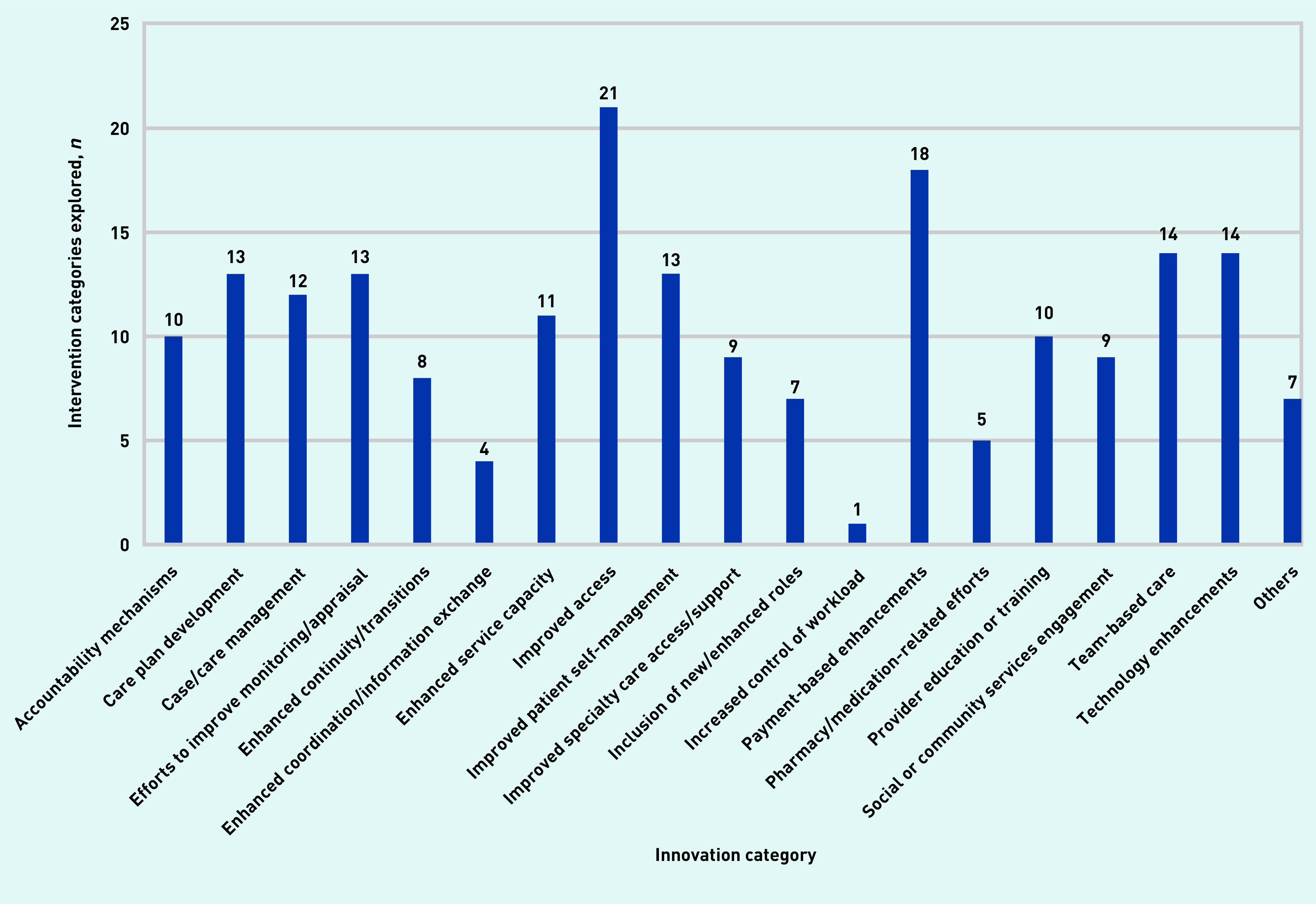
**Number of innovation categories included in studies. Categories are not mutually exclusive.**

The number of intervention categories explored in the included articles are presented in Figure 3. The most commonly studied types of innovations were those aimed at improving access (explored in 21 articles), followed by payment-based enhancements (in 18 articles), and innovations implementing team-based care or technology enhancements (each in 14 different articles). Conversely, innovations related to pharmacy/medication improvements, those trying to enhance coordination or information exchange, and those aimed at increasing the control over workload were studied in five, four, and one article respectively (Supplementary Table 2a–d).

The innovation categories explored in each of the included articles are outlined in Supplementary Tables S2a–d, with detailed descriptions of the innovations and the numerical magnitudes of their effects on the corresponding quadruple-aim outcome(s), given in Supplementary Table S4. The 4C that was most consistently aimed at to be improved was comprehensiveness — this featured in 34 of the 37 articles. Next in line came the interventions that aimed to have a positive impact on continuity (28 articles), first contact (23 articles), and coordination (20 articles). The description of the general direction of the effects of the innovations is presented, organised by the number of ‘Cs’ on which the studies had an impact (Supplementary Tables S2a–d and Supplementary Table S3).

Thirty-five per cent (13/37)^17^^–^^29^ of the interventions/programmes impacted all of the 4Cs (Supplementary Table S2a). These programmes included, on average, 7.25 intervention categories each (median = 7), and the most recurrently explored outcome was healthcare costs and utilisation (which featured in 10 of the 13 articles), followed by population health outcomes (in five of the 13 articles). Patient satisfaction was reported in four of these interventions, and one looked at provider satisfaction.

Programmes impacting on all of the 4Cs showed mixed results in almost all of their reported outcomes (Supplementary Table S2a). For utilisation, some parameters improved (increased screening and preventive services, increased visits to primary care relative to specialists) while, for other parameters, there were statistically significant and non-significant reductions in emergency department (ED) visits, outpatient visits, and hospitalisations. For expenditures, some studies reported cost savings and decreased costs, others reported no significant changes, and one study reported significant increases in costs for coverage of prescriptions.

For population health outcomes, though statistically significant improvements were reported for chronic illness care, patients with cardiovascular disease, control of diabetes, and the reduction in diabetes complications, there were also non-statistically significant changes for some diabetes parameters, control in patients with chronic obstructive pulmonary disease and asthma, vascular complications, and mental health. Two of the four interventions reporting on patient satisfaction found statistically significant improvements for perception of GPs’ work, while the other two reported mixed results for perceived quality and coordination of services, and no differences for access to care, same-day appointments, and satisfaction with primary care. The study looking at provider satisfaction also reported mixed results (significant increase in satisfaction for communication with patient and management of chronic care; no differences for overall satisfaction, knowledge of patients, and care coordination).

Nineteen per cent (seven out of 37) of the innovation programmes aimed to have an impact on three of the 4Cs (Supplementary Table S2b). These programmes averaged 5.6 intervention categories each (median = 6); six of seven studies explored healthcare costs and utilisation, and four of seven studies looked at population health outcomes. None explored patient or provider satisfaction.

The three programmes impacting on first contact, comprehensiveness, and continuity^30^^–^^32^ showed mixed results for population health (improvements for patients reaching low-density lipoprotein target levels; non-significant changes for quality-of-life scores) and utilisation outcomes (increased primary care annual services and visits; specialty visits remained the same, but the proportion of patients readmitted, along with the number of readmissions and hospital days, increased).

The two interventions that impacted comprehensiveness, continuity, and coordination^33^^,^^34^ also reported mixed results: these included a statistically significant lower risk of death, but non-statistically significant changes in health status and, for utilisation outcomes, a decrease in the number of specialist visits, but non-significant changes for hospital use and Medicare payments.

The study that impacted first contact, continuity, and coordination^35^ reported statistically significant increases in non-urgent primary care visits, but mixed results for hospital admission and length of stay, and a statistically significantly increased number of prescriptions. The study that impacted first contact, comprehensiveness, and coordination^36^ reported statistically significant improvements for diastolic blood pressure and microalbumin:creatinine ratio tests, but no significant changes for haemoglobin A1c, lipid measures, and the number of tests and ED/hospital visits.

Forty-one per cent (15 out of 37) of the innovation programmes impacted two of the 4Cs (Supplementary Table S2c). These programmes included an average of 4.1 intervention categories each (median = 4), and reported mostly on healthcare costs and utilisation outcomes (12 of 15 studies), followed by population health outcomes (five of the 15 studies). Two reported patient satisfaction outcomes and two provider satisfaction.

For the seven studies that impacted on comprehensiveness and continuity,^37^^–^^43^ there were mixed results for: population health outcomes (significant improvements in several diabetes measures, blood pressure control, and smoking status; no differences for other diabetes-related measures and cardiovascular health) and utilisation outcomes (improvements for screening services and more services provided, especially in capitation-based models; non-statistically significant changes for continuity of care, ED use, and several documentation parameters).

There were improvements in satisfaction for providers whose payment model factored in panel size. The three studies that impacted comprehensiveness and coordination^44^^–^^46^ reported: improvements in older populations for depression and dyspnoea, but no changes for other behavioural disorders, pain and falls; improvements for unplanned hospitalisations and increased preventive measures, and cost avoidance and decreased service utilisation for chronic conditions in incentive-based services. There were no changes in total hospital admissions, and increased costs for incentive-based diabetes services.

The three studies that focused on first contact and comprehensiveness^47^^–^^49^ reported mixed results for healthcare costs and utilisation — namely, statistically significant decreases in Medicare expenditures, per-member and per-quarter costs, and decreased primary care visits and visits per full-time equivalent, but no changes for hospitalisations, ED visits, and other utilisation outcomes. These studies reported statistically significant improvements for some patient satisfaction outcomes (timely appointments and self-management support, satisfaction with ability to see personal doctor, ease of getting care, and ratings of health care) but not for others (communication with providers, or knowledge of providers of other services). For providers, there was improved perception towards quality and services provided.

The two studies that impacted first contact and continuity^50^^,^^51^ reported mixed results for healthcare costs and utilisation outcomes (reduction of avoidable long-term ED visits, decreases in cost of drug prescriptions, increased GP consultations, and decreased specialist consultations, among others; increased costs of GP and specialist consultations, and no changes of ED hospitalisations).

Only two interventions^52^^,^^53^ impacted on just one of the 4Cs (comprehensiveness) (Supplementary Table S2d). These had one and two intervention categories, and reported mixed results for population health (some improvements in BP control for some patient groups but not for others) and resource utilisation outcomes (reductions for specialty care visits but non-statistically significant effects on proportion of patients seeing multiple doctors or surgical admissions).

## DISCUSSION

### Summary

To the authors’ knowledge, this is the first study summarising the impact of multicomponent interventions implemented internationally with the aim of enhancing primary care. Through this review, it was possible to identify which innovation features were most commonly included, how these interventions impacted the primary care core functions, and at which types of outcomes interventions were most consistently aimed. Overall, most of the articles presented statistically significant improvements for some of the measured outcomes while also presenting non-statistically significant changes or mixed results. No study presented only statistically significant improvements for all of the outcomes examined; this might be explained by the variety of elements included in each intervention (some having positive impacts, while others were neutral) and by the varied populations involved (for example, populations with disease improved more than general populations).

In terms of types of innovations, most of the efforts aimed at increasing access to primary care services by offering after-office and weekend hours, improving ease for scheduling appointments, and providing different access modalities (for example, telephone appointments, home visits). The second most common type of innovation was payment-based enhancements, such as financial incentives for providers to achieve some performance measures, comparisons of fee-for-service versus capitation models, additional payments for providers treating patients with complex healthcare needs, and changing a compensation formula to a salary based on panel size. Additional common innovations included providing team-based primary care, introducing technology-based enhancements, and supporting patient self-management.

The most common purpose of the interventions was to improve healthcare costs and/or utilisation outcomes, followed by interventions trying to improve population-health outcomes. Few articles explored the impact of their interventions on patient or provider satisfaction.

Improving primary care has been identified as one solution for improving population health outcomes, while maintaining or decreasing healthcare costs and resource utilisation. As a result, several countries have implemented multicomponent interventions aimed at enhancing policies or the way in which primary care services are provided. The evidence from the studies evaluating these efforts shows that most of these produce some benefits, especially in terms of increasing primary care visits relative to specialist visits, increasing screening and preventive services, and improving patient and provider satisfaction. However, at the same time, they also present non-statistically significant results depending on the individual features of the innovations, the outcome(s) being measured, and the populations being studied. Further research is needed to identify the interventions — alone or in combination — that would produce consistent benefits for various populations, policy environments, and health system structures. This will require systematic, context-sensitive assessment of the relationship between the specific interventions intended to enhance the functional goals of primary care and the degree to which these changes achieve those goals and, in turn, achieve optimal population health.

### Strengths and limitations

The main strength of this study is that real-world efforts to enhance primary care were examined; these commonly involved several innovation features being implemented simultaneously, as well as their impact on both primary care functions and outcome measures. A large number of studies explore the impact of a single innovation, which may overlook real-world settings, in which there may be interactions with other concurrent innovations or contextual factors. Additionally, these studies usually aim to connect the intervention directly to an outcome, overlooking the impact on primary care core functions that may act as a process measure for ‘how’ an innovation may impact outcomes. In this study, the authors identified the need to connect the innovation with the primary care core function, which would eventually lead to the desired outcome.

The review does have some limitations that should be acknowledged. Given the large number of studies found using one health-related online search engine and database (that is, Ovid MEDLINE), the authors did not search other electronic databases. However, the number and characteristics of the retrieved studies was sufficient for the purpose of the review and, though using one medical electronic search engine and database may overlook studies in other relevant databases, MEDLINE, the US National Library of Medicine’s premier bibliographic database (https://www.nlm.nih.gov/bsd/medline.html) was selected — this is, arguably, the most comprehensive database, containing in excess of 25 million records. In addition, to supplement this, a manual search of the references of the included studies was conducted, as was a grey-literature search, to look for additional articles that might be relevant.

Another potential limitation relates to the specific inclusion criteria. As described, the most relevant studies were those that included interventions with several concurrent innovations that not only reported outcomes, but also provided numerical values for the impact on those outcomes. This may omit important evidence from single-element interventions or from studies that did not report outcome results numerically, though a scan through the Tier 2 studies did not suggest a failure to identify major strategies for enhancing primary care. Additionally, given that interventions present a mix of innovations, it was difficult to elucidate which element was responsible for changes in a particular outcome measure; in contrast, in studies that looked specifically at one type of innovation, an outcome could be directly linked to that innovation. However, the focus of the review was to identify how a primary care ‘innovation environment’ comprising several different elements could improve or enhance the way primary care was provided and, possibly, its potential effect on outcomes.

It is important to mention that, though most of the analysed studies here stem from developed high-income countries, none was from the UK, as they did not completely fulfil the authors’ strict study eligibility requirements. The electronic database search did retrieve articles from the UK but, given that these reported on more specific interventions and provided fewer outcome measures, they were classified as Tier 2 articles and, thus, not included in the analysis.

Additionally, the conflicting results presented here may reflect the inclusion of interventions from different health systems with different cultural and political influences; a possible next step, therefore, could be to look at specific countries with more homogeneous cultures and models of care (for example, the US, where there is some commonality in the system of healthcare delivery), to ascertain whether more consistent results are found.

### Comparison with existing literature

These multicomponent interventions re-emphasise the point made by Corscadden *et al*^54^ and Doran *et al*^55^ that the main perceived barriers to enhanced primary care relate to patients having difficulties accessing primary care services and providers not having the right type of incentives to provide better-quality services. Additionally, the frequent focus on improvements aimed at supporting patient self-management and providing team-based care reflect the increasing recognition of the importance of the patient becoming a partner in their own health care,^56^ and a growing consensus that an ideal way to deliver services at primary care level involves a team of providers, who are able to follow up and frequently engage with the patient.^57^ As seen at other levels of care, this study identified an important number of interventions that explored the role that technology could play in improving primary care services,^58^ which is consistent with the published literature. The technologies identified here were mostly related to enhancements of electronic medical records, data-monitoring systems, and electronic health registries to identify at-risk patients.

The most common outcomes explored in these innovation environments — that is, population health and cost/utilisation outcomes — correspond with the literature reporting that many countries seeking to strengthen their primary care systems focus mainly on improving the health of the population and on reducing healthcare costs.^59^^–^^61^

### Implications for research and practice

From the very general trends identified here, it seems that, on the whole, interventions that have continuity as a commonly impacted core function increase primary care consultation, especially relative to the maintenance or decrease in specialist visits. Also, generally, there are consistent improvements for screening and/or preventive services, which were most commonly associated with interventions categorised as impacting on comprehensiveness. Overall, in the few studies that reported on satisfaction, it seems that most efforts are associated with improvements for both patient and physician, for example, improvements aimed at first contact correlated with improved satisfaction of patients with appointments and ease of getting care. Thus, it is important for future research to be able to identify which primary care core function (that is, ‘C’) is the one to aim at in order to improve a specific outcome.

In contrast, the outcomes most consistently obtaining mixed results relate to hospital admissions and ED visits, and expenditures. No matter which, or how many, of the 4Cs were affected by the interventions, these outcomes showed either statistically significant improvements and deteriorations simultaneously, or non-statistically significant changes. Again, it is important for future research to be able to connect resource or utilisation measures to a primary care core function, in order to clearly identify how these type of measures could be improved.

What is notable is the heterogeneity of this literature. This is reflected not only in terms of the interventions applied, but also in the: inconsistency in evaluating contextual features, such as policy environment and sociodemographics; lack of clarity of the proposed causal relationship between innovations, process changes, and ultimate outcomes; and in the variable tracking of process and outcomes. To support more consistency in future studies, a conceptual framework, such as that illustrated in Figure 1, could provide a useful starting point. Finally, the increasing importance of improving primary care is reflected in the number of studies reporting on interventions or programmes aimed at enhancing primary care services in some way. Given that most of the interventions presented here were responsible for improving some aspects of primary care but not others, or improving measures for some populations but not for others, there is a clear need for further studies to determine how a multicomponent primary care intervention could more consistently improve a wider range of primary care aspects, and for more of these studies to explore satisfaction outcomes for patients and providers.
